# Discovery of Novel Serum Biomarkers for Prenatal Down Syndrome Screening by Integrative Data Mining

**DOI:** 10.1371/journal.pone.0008010

**Published:** 2009-11-24

**Authors:** Jeroen L. A. Pennings, Maria P. H. Koster, Wendy Rodenburg, Peter C. J. I. Schielen, Annemieke de Vries

**Affiliations:** 1 Laboratory for Health Protection Research (GBO), National Institute for Public Health and the Environment (RIVM), Bilthoven, The Netherlands; 2 Laboratory for Infectious Diseases and Perinatal Screening (LIS), National Institute for Public Health and the Environment (RIVM), Bilthoven, The Netherlands; Leeds Institute of Molecular Medicine, United Kingdom

## Abstract

**Background:**

To facilitate the experimental search for novel maternal serum biomarkers in prenatal Down Syndrome screening, we aimed to create a set of candidate biomarkers using a data mining approach.

**Methodology/Principal Findings:**

Because current screening markers are derived from either fetal liver or placental trophoblasts, we reasoned that new biomarkers can primarily be found to be derived from these two tissues. By applying a three-stage filtering strategy on publicly available data from different sources, we identified 49 potential blood-detectable protein biomarkers. Our set contains three biomarkers that are currently widely used in either first- or second-trimester screening (AFP, PAPP-A and fβ-hCG), as well as ten other proteins that are or have been examined as prenatal serum markers. This supports the effectiveness of our strategy and indicates the set contains other markers potentially applicable for screening.

**Conclusions/Significance:**

We anticipate the set will help support further experimental studies for the identification of new Down Syndrome screening markers in maternal blood.

## Introduction

For over two decades, prenatal screening for Down Syndrome (DS) has been available to pregnant women. A screening procedure usually consists of a risk calculation based on maternal serum measurements and other parameters like nuchal translucency and maternal age, after which women with a high predicted risk can opt for invasive testing such as amniocentesis or chorion villus sampling. Initially, the most commonly used method for risk calculation was the second trimester triple test, which combines serum levels for alpha-fetoprotein (AFP), unconjugated estriol (uE3), and the free β subunit of human chorion gonadotrophin (fβ-hCG) with maternal age [1], [2]. In recent years, this test has been largely replaced by the first trimester combined test, which is based on fβ-hCG and pregnancy-associated plasma protein A (PAPP-A) serum concentrations, ultrasound nuchal translucency (NT) measurements and maternal age [3]. The latter test is the method currently available to pregnant women in the Netherlands.

Despite international experimental effort to improve the DS screening, both the Detection Rate (DR) and False Positive Rate (FPR) can still significantly be improved upon. In the Netherlands, the current DS screening has a DR of 75.9% and an FPR of 3.3% [4]. Most research effort in this field is focused on finding new biomarkers for which serum levels can be added to the risk calculation algorithm. In recent years, proteomics methods for large-scale protein quantitation have been employed to facilitate the search for such biomarkers [5]–[10]. However, the performance of candidate biomarkers obtained by such studies are not always reproducible, and also established DS pregnancy biomarkers are not always successfully confirmed in such studies, likely due to issues related to technical sensitivity and reproducibility.

A recent study by our group used bead-based multiplexed immunoassays to test 90 different analytes in first trimester maternal serum samples for DS pregnancies and controls [7]. This study identified seven new potential biomarkers that allow for a more accurate first trimester risk prediction, while confirming the long-known usefulness of PAPP-A. The set of 90 analytes was not pregnancy- or DS- specific but based on a pre-fixed commercially available set. We reasoned that with a set that is more focused on markers relevant for pregnancy or DS, more and also more specific biomarkers can be found. Hence, we set out to develop such a set by analysis and integration of publicly available data.

The amount of information on genes and proteins in databases is increasing rapidly, which allows for a bioinformatics approach that involves automated collecting and combining information from biological databases, known as data mining. Recent studies using data mining for identification of blood based cancer biomarkers showed the successfulness of this approach [11], [12].

Current DS screening protein biomarkers can be traced to originate from two tissues, namely fetal liver (e.g. AFP) and the placenta (e.g. fβ-hCG, PAPP-A). The non-protein serum biomarker uE3, routinely used in 2^nd^ trimester screening, is produced by the placenta from its precursor dehydroepiandrosterone sulfate derived from the fetal adrenal glands and liver [13], [14]. Placental markers can be assigned more specifically to the trophoblast cells, which are involved in both the implantation of the embryo into the endometrium as well as the production of hormones required for establishing and sustaining pregnancy. Indeed, abnormal trophoblast differentiation has been observed in placentas of DS pregnancies [15], [16]. As the current screening biomarkers are all derived from the two tissues mentioned (i.e. fetal liver and placental trophoblast cells), we hypothesized that several novel useful biomarkers can primarily be found to be derived from these two tissues. To identify such protein biomarkers we combined data from several publicly available sources.

## Methods

### Analysis of Tissue-Specific Gene Expression Data

Human tissue-specific gene expression data were analyzed using the Symatlas web-interface (http://symatlas.gnf.org) based on data previously published by Su et al [17], [18]. Symatlas data were considered most useful for this study as it provides the largest publicly accessible data collection on multiple tissues, including both adult and fetal tissues. Tissue data used were both from the Human GeneAtlas GNF1H (gcRMA-normalised) (79 tissues) and the Human GeneAtlas U95A (44 tissues). Using the web-interface, these data sets were queried for the number of genes highly expressed in fetal liver or placenta, according to different stringency levels. Such a stringency level consists of a minimal ratio for the gene expression in a target tissue (in our case placenta or fetal liver) compared to the median expression of that gene across all tissues examined. Using various stringency levels, the number of tissue-specific genes obtained for each stringency level was determined for fetal liver as well as placenta. The resulting data were imported into the statistical program R (www.r-project.org) [19] and the data distribution was assessed to determine the nonspecific underlying trend over lower stringency levels. This revealed that for lower thresholds this trend could be approximated with a power law distribution, where a two-fold increase in the threshold led to a four-fold decrease in the number of genes expressed above that threshold. We refer to this trend as the nonspecific underlying trend. For higher stringencies, the number of genes began to decrease at a slower rate, indicating an enrichment for tissue-specific genes over the nonspecific trend. Based on this finding, a threshold was set that yielded approximately 10 times more tissue-specific genes than could be estimated based on the nonspecific underlying trend. In other words, using the trend for nonspecific genes at lower stringency levels, a stringency threshold was chosen that was high enough to consider 90% of the genes highly expressed in either fetal liver or placenta to be specifically derived from that tissue and not be a statistical artefact. These genes were used in subsequent analysis steps. The same approach was used to enrich fetal-liver-specific genes compared to adult liver-specific genes.

### Text-Mining

As text-mining is still a developing field, we wanted to include more than one text-mining tool to restrict the chance of false negatives. For that reason two applications were combined as they use different approaches to search partially different databases, and therefore can be considered complementary. The first of these is Anni (http://www.biosemantics.org/anni/) [20], which provides an ontology- and thesaurus-based interface to Medline and retrieves associations for several classes of biomedical concepts (e.g. genes, drugs, and diseases). These concepts are given a concept weight, which indicates their relevance to the applied search term. The second application is Polysearch (http://wishart.biology.ualberta.ca/polysearch) [21], which supports different classes of information retrieval queries against several different types of text, scientific abstract or bioinformatic databases such as PubMed, OMIM, DrugBank, SwissProt, the Human Metabolome Database (HMDB), the Human Protein Reference Database (HPRD), and the Genetic Association Database (GAD). The relevancy scores of the obtained genes or proteins are expressed as Z scores, *i.e.* as standard deviations above the mean. The two applications were searched for genes associated with the terms “trophoblast”, “cytotrophoblast”, and “syncytiotrophoblast”. Significance criteria for Anni were based on a minimally tenfold enrichment over the statistically determined distribution of the concept weight. For PolySearch, a Gaussian distribution was used, based on the software documentation. Gene lists obtained for the three terms were combined and subsequently manually adjusted to resolve ambiguous or redundant gene symbols.

### Assessing Applicability for Blood-Based Detection

To determine if putative biomarkers identified by gene expression analyses and/or text mining are potentially blood-detectable, they were cross-checked against two different data resources. Proteins were considered blood-detectable if they had at least one of the Gene Ontology (GO) annotation terms “extracellular region”, “extracellular region part”, or “extracellular space”; or if they were included in the Human Plasma Proteome (HPP) list. GO (http://www.geneontology.org) [22] annotations are partially based on computational predictions whereas the HPP list [23] is based on a combination of experimental methodologies. The latter approach revealed some blood-detectable proteins not predicted by Gene Ontology, but lacked some low-abundance proteins including protein hormones [23]. As with the text-mining tools, these approaches were therefore considered complementary and results were combined.

## Results

### Identification of Tissue-Specific Candidate Genes

**Figure 1:**
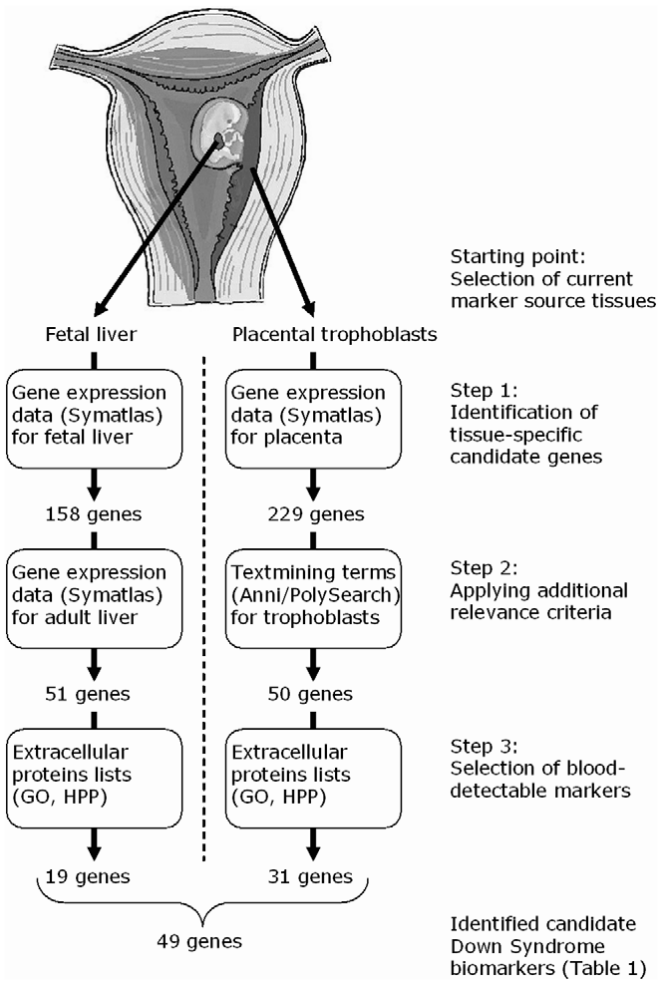
Schematic representation of the steps employed in our data mining strategy and the number of genes selected after each step.

The DS screening biomarkers currently implemented in the first trimester combined test or the second trimester triple test are derived from two tissues, namely fetal liver and placental trophoblasts. Therefore, the first step in our data mining approach consisted of identifying genes specifically expressed in either one of these two tissues (see Fig. 1 for an overview of the various selective steps).

The tissue-related gene expression resource Symatlas was searched for genes expressed in either fetal liver or placenta, at a level that is a (user-definable) multiple of the median expression for that gene across all tissues. By using various threshold levels and statistical analysis, we found that at a gene expression threshold of 30 times the median tissue expression, ten times more genes were identified by the Symatlas query than were expected based on the nonspecific trend. This applied to fetal liver as well as placenta. Therefore, we used this criterion to select genes that have a high probability of being specific for these individual tissues. This approach resulted in 158 proteins specific for fetal liver and 229 for placenta, respectively (Fig. 1).

### Applying Additional Relevance Criteria

The second step consisted of further prioritizing our set of genes by ensuring that the genes selected in step 1 are not only highly expressed, but are also sufficiently relevant for the tissues mentioned. In the case of fetal liver-specific genes, we again used Symatlas to ensure that the expression in fetal liver exceeded at least ten times that of adult liver, thus narrowing down the list from 158 to 51 genes (Fig. 1). For placenta-specific genes we used two complementary textmining tools (Anni and Polysearch) to select genes related to three trophoblast-related search terms (trophoblast, cytotrophoblast, syncytiotrophoblast). For Anni, genes with a concept weight >0.0001 (based on yielding ten times more terms than expected for a nonspecific distribution) and for Polysearch genes with a Z value>1.6 for at least one of the terms were selected. This way, 181 genes were found, 50 of which were also found to overlap with the previous selection of 229 placenta-specific genes (Fig. 1). We applied a different filtering method for the two tissues because Symatlas does not include gene expression data specific to trophoblasts or its two subtypes, whereas text mining was less able to make a distinction between proteins related to fetal or adult liver.

The 51 fetal liver-specific genes, and 50 trophoblast-related genes were subsequently analyzed for detectability in blood.

### Selection of Blood-Detectable Markers

For implementation of a biomarker in a routine human screening program, it is essential that it can be detected in serum or plasma. For the markers selected by the previous steps, we examined which ones had a Gene Ontology annotation as being extracellular, or were part of the experimentally derived Human Plasma Proteome list compiled by Anderson *et al*. [23]. This final selection step resulted in 49 individual blood-detectable markers (Fig. 1, Table 1). For fetal liver and placenta, these numbers were 19 and 31, respectively, with IGF2 being part of both sets (Fig. 1, Table 1).

**Table 1:** Identified candidate Down Syndrome (DS) biomarkers.

Symbol	Chrom	Description (synonym)	Potential
*Fetal liver-derived markers*			
**AFP**	4	alpha-fetoprotein	In use
ANGPTL3	1	angiopoietin-like 3	
C5	9	complement component 5	
COL1A1	17	collagen, type I, alpha 1	Indications
COL1A2	7	collagen, type I, alpha 2	
COL2A1	12	collagen, type II, alpha 1	
COL3A1	2	collagen, type III, alpha 1	Indications
COL5A2	2	collagen, type V, alpha 2	
DEFA3	8	defensin, alpha 1	
DLK1	14	delta-like 1 homolog	
ELA2	19	elastase 2, neutrophil	
GPC3	X	glypican 3	
IGF2	11	insulin-like growth factor 2 (somatomedin A)	
PF4	4	platelet factor 4 (CXCL4)	
PPBP	4	pro-platelet basic protein (CXCL7)	
RRM2	2	ribonucleotide reductase M2 polypeptide	
S100A8	1	S100 calcium binding protein A8 (calgranulin A)	
S100A9	1	S100 calcium binding protein A9 (calgranulin B)	
SPTA1	1	spectrin, alpha, erythrocytic 1	
*Placental trophoblast-derived markers*			
ADM	11	adrenomedullin	
ALPP	2	alkaline phosphatase, placental	
CDH1	16	cadherin 1, type 1, E-cadherin	
CDH11	16	cadherin 11, type 2, OB-cadherin	
CGA	6	glycoprotein hormones, alpha polypeptide	
**CGB5**	19	chorionic gonadotropin, beta polypeptide (**βHCG**)	In use
CRH	8	corticotropin releasing hormone	
CSH1	17	chorionic somatomammotropin hormone 1 (placental lactogen)	Biomarker
CSH2	17	chorionic somatomammotropin hormone 2	Biomarker
EBI3	19	epstein-barr virus induced gene 3	
EGFR	7	epidermal growth factor receptor	
FN1	2	fibronectin 1	
GH1	17	growth hormone 1	
GH2	17	growth hormone 2, placenta-specific growth hormone	Biomarker
IGF2	11	insulin-like growth factor 2 (somatomedin A)	
IGFBP1	7	insulin-like growth factor binding protein 1	Examined
INHA	2	inhibin, alpha	Biomarker
INHBA	7	inhibin, beta A (activin A, activin AB alpha polypeptide)	Examined
INSL4	9	insulin-like 4	
LGALS13	19	lectin, galactoside-binding, soluble, 13 (PP13)	Examined
**PAPPA**	9	pregnancy-associated plasma protein A, pappalysin 1	In use
PGF	14	placental growth factor	Biomarker
PLAC1	X	placenta-specific 1	
PLAU	10	plasminogen activator, urokinase	
PRL	6	Prolactin	
PSG5	19	pregnancy specific beta-1-glycoprotein 5	
SERPINB2	18	serpin peptidase inhibitor, clade B, member 2	
SERPINE1	7	serpin peptidase inhibitor, clade E, member 1 (PAI1)	
SPP1	4	secreted phosphoprotein 1 (osteopontin)	
TGFB1	19	transforming growth factor, beta 1	
TIMP3	22	TIMP metallopeptidase inhibitor 3	

Potential for DS screening is indicated as follows: In use, currently widely used in 1^st^ or 2^nd^ trimester DS screening (in bold); Biomarker, studies showed overall significant concentrations; Examined, examined as biomarker but not significant or inconclusive overall results; Indications, found in high-throughput study but awaiting further study. References on the corresponding literature are given in the Discussion.

## Discussion

The aim of this study was to design a set of new potential blood-detectable biomarkers for prenatal DS screening by computational data mining, that is more focused on DS screening than currently available commercial multiplex kits or high-throughput methods for whole proteome analysis. By combining data from different (publicly available) data sources into a three-stage approach (summarized in Fig. 1), we identified 49 of such protein markers (Table 1). Our combined list contains three biomarkers that are currently widely used in either first- or second-trimester DS screening, namely AFP, PAPP-A and fβ-hCG. This demonstrates that the method used is able to identify relevant DS screening biomarkers. In addition, the list contains several other proteins which have been examined for their potential as DS screening biomarkers by several research groups, such as the inhibin chains INHA and INHBA [24]–[27], the (protein-identical) placental lactogen genes CSH1 and CSH2 [28], placental growth hormone (GH2) [29]–[31], placental growth factor (PGF) [32], IGFBP1 [30], or PP13 (LGALS13) [33]. For five of these proteins (INHA, CSH1, CSH2, GH2, PGF), significant differences in concentration exist between DS and euploid pregnancies, and therefore these can be used as a biomarker in DS screening. Additionally, two collagen-related markers, COL1A1 and COL3A1, (as well as IGFBP1) have been described to have different amniotic fluid levels in DS pregnancies [10] and it is conceivable that this also applies to the corresponding maternal serum levels although this remains to be established.

While identifying AFP, PAPP-A as well as fβ-hCG as DS screening biomarkers, our approach failed to identify the second trimester biomarker unconjugated estriol (uE3). However, as uE3 is not a protein biomarker, it is not supported by our strategy based on gene expression and protein data integration. Another biomarker that our approach failed to detect but has been described in the literature is ADAM12. This protein is both highly expressed in placenta and extracellular, but failed the criteria used in the textmining step. It should be noted, however, that most recent studies find this biomarker to be informative only before 10 weeks of gestation, so the applicability of this protein is already limited [34]–[38]. The finding that two complementary textmining methods did not find sufficient evidence for association of ADAM12 with trophoblasts can either indicate that current literature databases only provide weak evidence for this association, or that both textmining tools were not successful in detecting an existing association. As textmining is a developing field, both options are plausible. A recommendation for future studies of this kind might therefore be to consider including further textmining tools based on additional search algorithms.

Among the 49 proteins in Table 1, several overrepresentations of biological processes can be observed. Among the fetal liver-derived genes the five collagen genes are most apparent, but there are also a number of proteins related to innate immunity such as C5, PF4, PPBP, S100A8, and S100A9. These immunological proteins can be ascribed to the central role the fetal liver has in fetal hematopoiesis. For the placental trophoblast-derived proteins the majority act as hormones or growth factors, and in addition four proteins (PLAU, SERPINB2, SERPINE1, TIMP3) are involved in tissue remodeling. Both these processes are associated with the role of placental trophoblasts in the production of hormones required for establishing and sustaining pregnancy as well as the implantation of the embryo into the endometrium. Given that most of the identified markers are associated with a small number of biological processes, it becomes likely that these pathways might also harbor other potential DS screening markers that do not meet the criteria used in our approach or for which insufficient data are available.

As Down syndrome is caused by a (partial) trisomy of chromosome 21, it seems counterintuitive that none of the markers in Table 1 are located on chromosome 21. Although it might be expected that genes on this chromosome are expressed at an approximately 1.5-fold higher level compared to other genes and could therefore act as suitable biomarkers, this assumption does not fully hold in comparative studies [39]–[41]. Furthermore, proteomic studies including our own found no increased presence of chromosome 21 proteins among the differentially expressed proteins [5]–[10]. Moreover, although partial trisomy 21 is sufficient for DS, efforts to associate DS with a smaller chromosomal region have excluded the possibility of a single region being responsible for all aspects of the phenotype [42]–[45]. Additionally, several characteristics of a DS phenotype are also found for other types of aneuploidy, indicating that the higher expression of genes located on chromosome 21 is only linked indirectly to DS phenotype and mainly acts through disregulation of genes on other chromosomes. This can also explain why current DS screening biomarkers are not located on chromosome 21 and the pregnancy screening biomarkers in use are also predictive for other chromosomal aberrations such as Edwards syndrome (trisomy 18) and Patau syndrome (trisomy 13). This actually creates the possibility that some of the markers mentioned in Table 1 are not only applicable to DS screening, but also to pregnancies with other types of fetal aneuploidy.

By means of integrative data mining we have derived a set of candidate Down Syndrome screening biomarkers. As the first two filtering steps are both based on a minimally ten-fold enrichment or induction over the corresponding background, we expect the number of false positives, i.e. not relevant markers, to be low. This is corroborated by the presence of eight proteins in our set that are in use or can be used as biomarker for DS screening and five other proteins for which this has been studied. However, before biomarkers can be tested in a large-scale cohort study, additional serum analysis experiments will be necessary to validate which of these candidate biomarkers have differential levels in DS versus normal pregnancies. Furthermore, we cannot predict beforehand at what gestational age biomarkers are most discriminatory between normal and DS pregnancies, and as a result, whether they can be integrated in late first or early second trimester screening. If this proves not to be the case, the usability of the new biomarkers in a routine, large-scale population screening program as applied in The Netherlands will be rather low. These further experimental validations of the new DS screening biomarkers identified by our data mining approach will evidently be the subject of future follow-up studies.
